# Associations of the *eNOS* G894T gene polymorphism with target organ damage in children with newly diagnosed primary hypertension

**DOI:** 10.1007/s00467-015-3164-9

**Published:** 2015-08-01

**Authors:** Joanna Śladowska-Kozłowska, Mieczysław Litwin, Anna Niemirska, Aldona Wierzbicka, Marta Roszczynko, Małgorzata Szperl

**Affiliations:** Department of Nephrology and Arterial Hypertension, The Children’s Memorial Health Institute, Warsaw, Poland; Department of Biochemistry and Experimental Medicine, The Children’s Memorial Health Institute, Warsaw, Poland; Department of Molecular Biology, Institute of Cardiology, Warsaw, Poland

**Keywords:** Primary hypertension, Children, Intima media thickness, *eNOS* G894T gene polymorphism, Target organ damage, Nitric oxide

## Abstract

**Background:**

The endothelial nitric oxide synthase (*eNOS*) G894T gene polymorphism is associated with the risk of primary hypertension (PH) and vascular complications in adults with PH.

**Methods:**

We explored the associations of the G894T polymorphism with 24-h ambulatory blood pressure, left ventricular mass (LVM), carotid intima media thickness (cIMT), urinary albumin excretion, oxidative stress and inflammatory parameters in 126 children with newly diagnosed PH and in 83 healthy children.

**Results:**

Among the 126 children with PH 92 (73 %) had ambulatory hypertension and 34 (27 %) had severe ambulatory hypertension. Left ventricular hypertrophy (LVH) was detected in 39 (31 %) patients, cIMT of >2 standard deviation scores in 21 (16.6 %) patients, albuminuria of >30 mg/24 h in 18 (14.3 %) patients and metabolic syndrome (MS) in 22 (17.5 %) patients. The frequency of the T allele was 52.4 % in the PH group and 54.2 % in the control group (not significant), and in both groups the frequency of the T allele was consistent with the Hardy–Weinberg equilibrium. Compared with G allele carriers, hypertensive T allele carriers had increased cIMT (*p* < 0.05) and more severe albuminuria (not significant, *p* = 0.1); there was no difference between the groups in hypertension severity and LVM. T and G allele distribution did not differ between patients with and without metabolic syndrome. No significant correlations between the assessed parameters and the *eNOS* G894T gene polymorphism were found in the controls, although T allele carriers tended to have an increased cIMT (*p* = 0.09).

**Conclusion:**

The *eNOS* T allele is not more prevalent among hypertensive children than among healthy ones, but it is associated with early vascular damage in children with PH, independent of metabolic abnormalities. No associations between the *eNOS* G894T polymorphism and metabolic abnormalities were found.

## Introduction

Subclinical target organ damage (TOD) in the form of left ventricular hypertrophy (LVH) and/or increased carotid intima-media thickness (cIMT) is already present in 30–40 % of children with primary hypertension (PH) at the diagnosis of elevated blood pressure (BP) [1–5]. Several complex mechanisms have been hypothesized to play a key role in the development of vascular complications, including metabolic and inflammatory processes, as well specific genetic predisposition. Some data indicate the involvement of endothelial nitric oxide synthase (eNOS) in the development of PH and the association of a relative or absolute decrease of eNOS activity with various vascular complications in response to hemodynamic workload [6]. Other data indicate that relative or absolute defects in the production of nitric oxide (NO) by eNOS or an abundant degradation of NO by enhanced oxidative stress (reactive oxygen species) is associated with various vascular complications in response to hemodynamic workload [6, 7]. In addition, impaired NO bioavailability can also be related to a cellular defect in skeletal muscle tissue, where NO regulates many metabolic and contractile processes, such as basal glucose transport [8].

Several polymorphisms of the *eNOS* gene have been identified, including a single nucleotide polymorphism (SNP) in the promoter region (T^−789^C), a variable tandem repeat in intron 4 and a Glu298Asp SNP in intron 7. A glutamic acid to aspartic acid substitution at amino acid position 298 is caused by Guanine (G) to thymine (T) transversion at nucleotide 894 of exon 7 [9]. The *eNOS* 894 T variant has lower activity and has been found to associate with coronary heart disease, carotid atherosclerosis and endothelial dysfunction [10–17]. This variant is also associated with enhanced vasoconstrictive response to phenylephrine, hypertensive response to endurance training and development of hypertension [13, 18, 19]. However, it seems that both distribution and clinical relevance of the *eNOS* G894T polymorphism vary among different ethnic groups [20, 21].

To date, the relationship between the *eNOS* G894T gene polymorphism and vascular complications in hypertensive children has not been investigated. The main intermediate phenotype of children with PH includes metabolic abnormalities typical of metabolic syndrome, oxidative stress and immune activation. These abnormalities are strictly associated with TOD [22–25]. Although there are a few pediatric studies analyzing associations between *eNOS* polymorphisms and hypertension, obesity and metabolic syndrome, we are not aware of any pediatric study analyzing the associations of *eNOS* polymorphisms with hypertensive TOD in children with PH [26–28]. Thus, the aim of our study was to explore associations of the *eNOS* G894T gene polymorphism with TOD markers, oxidative stress and metabolic and inflammatory parameters in an ethnically homogenous group of 126 children with newly diagnosed PH and in 83 healthy children.

## Patients and methods

### Patients

White, Caucasian children and adolescents admitted consecutively to the The Children’s Memorial Health Institute during the period 2005–2008 for investigation of suspected arterial hypertension who had not been previously treated with antihypertensive drugs and in whom PH was ultimately diagnosed were eligible for entry into the study. Of these 200 children, 126 (median age 15 years, age range 5–18 years; 31 girls, 95 boys) with untreated PH who completed all investigative procedures were recruited to the study. PH was diagnosed after a thorough clinical and laboratory diagnostic work-up according to recently published recommendations [29]. In all patients the diagnosis was confirmed by 24-h ambulatory blood pressure measurement (ABPM). Recordings lasting at least 20 h with at least 80 % of records available for analysis were considered to be valid. BP values were calculated from the ABPM as the mean 24-h systolic BP (SBP/24 h) and diastolic BP (DBP/24 h) and presented as absolute values and as the SBP and DBP indices (SBPi and DBPi, respectively) calculated as the ratio of SBP or DBP to the 95th percentile for age and gender based on the pediatric ABPM reference data [30]. The recently published classification system based on ABPM was used to classify patients as having normal blood pressure, ambulatory hypertension and severe ambulatory hypertension [31]. The exclusion criteria were: diagnosis of secondary hypertension, previous use of antihypertensive drugs, presence of any significant chronic disease (except for PH) and acute disease, including infections, in the 6 weeks immediately preceding enrollment. Patients with incomplete biochemical data were also excluded.

### Controls

The control group consisted of 83 Caucasian healthy, normotensive children with a median age 12 (range 5–18) years (43 girls, 40 boys), recruited voluntarily from schools. None of these children had any significant chronic or acute disease, including infections, within the 6 weeks immediately preceding enrollment.

### Molecular studies

EDTA-anticoagulated venous blood samples were collected from all participating children. DNA was extracted from blood leukocytes using the phenol method. Polymorphism of *eNOS* G894T gene was evaluated by restriction fragment length polymorphism (RFLP) PCR.

### Anthropometrics and biochemical assays

All patients and controls underwent the following assessments, unless indicated otherwise, which included anthropometrical measurements, such as body mass index (BMI), waist circumference (WC) (patients only) and waist-to-hip ratio (WHR) (patients only). Obesity and overweight was diagnosed according to the International Obesity Task Force (IOTF) recommendations [32]. In all patients and controls, serum glucose and insulin, blood lipids, serum homocysteine, serum uric acid and C-reactive protein [high-sensivity C-reactive protein (hsCRP)], serum asymmetric dimethyloarginine (ADMA) levels, oxidative stress [thiobarbituric acid reactive substances (TBARS) and oxidized low-density lipoprotein cholesterol (oxyLDL) concentration] and anti-oxidative defense [reduced glutathione (GSH) concentration and glutathione peroxidase (GPx) activity] were measured. Because some of our patients were under 10 years of age, metabolic syndrome (MS) was diagnosed when at least three criteria were fulfilled [BMI ≥95th percentile for age and gender; arterial hypertension; serum triglycerides >110 mg/dl; fasting plasma glucose >100 mg/dl or >140 mg/dl at 2 h of oral glucose tolerance test; high-density lipoprotein (HDL) cholesterol <40 mg/dl] [22].

### cIMT measurements

The cIMT was measured both in controls and PH patients by ultrasound according to methodology described previously [3]. The median and standard deviation (SD) of normal values for cIMT were obtained from normative data published elsewhere [33].

### Echocardiography 

Echocardiography (ECHO) was performed in all patients, but due to organizational problems ECHO was not performed in controls. All ECHO examinations were performed by one examiner blinded to the severity of PH and effectiveness of treatment. ECHO measurements were performed according to the American Society of Echocardiography guidelines and to standardize the left ventricular mass to height ratio, the left ventricular mass index (LVMi) was calculated according to the de Simone formula [34]. LVH was defined as an LVMi value of >95th percentile for age and gender, based on the pediatric LVMi reference data [35].

### Laboratory investigations

Blood samples were taken after 12 h of fasting and immediately sent to the laboratory. Plasma glucose level was measured by a Siemens Dimension chemistry analyzer (Siemens Medical Solutions, Malvern, PA) . Plasma insulin concentrations and glycated hemoglobin (HbA1c) were measured by radioimmunoassay. An oral glucose tolerance test was carried out in all patients after oral ingestion of 1 g/kg body weight (maximum 75 g) of glucose. Insulin resistance, as described previously, was expressed as homeostasis model assessment for insulin resistance (HOMA-IR) and as the total triglycerydes:HDL-cholesterol (TTG/HDL) ratio.

The CRP concentration (hsCRP) was determined using highly sensitive immunoturbidimetry (Orion Diagnostica, Espoo, Finland). Lipids and uric acid (UA) concentrations were determined with standard laboratory procedures. Plasma lipid peroxides were determined with the spectrofluorometric method of Yagi and expressed as TBARS. GSH concentration and GPX activity were used as indicators of antioxidant status and were measured spectrophotometrically in erythrocytes (Oxis Bioxytech GSH-420 and GPx-340 assays, respectively; Oxis International Inc., Foster City, CA). OxyLDL and ADMA concentrations were measured by enzyme-linked immunosorbent assay (ELISA) using a commercially available kit. Urinary albumin excretion in 24-h samples was determined by the immunonephelometric method.

The laboratory procedures have been described in detail in a previous study [23].

### Statistics

Because the analyzed groups included subjects of different age and gender, BMI, WC and cIMT values were expressed both as absolute values and as standard deviation scores (SDS) for age and gender. LVM values (in grams) were standardized to height in meters^2.7^. Homogeneity of variance was checked with the Levene test. Continuous variables with a normal distribution were compared by the Student* t* test for independent variables and expressed as the mean and SD. Continuous values with a non-normal distribution were compared by the Mann–Whitney* U* test and expressed as the median and range. Comparison between groups was evaluated by the analysis of variance (ANOVA) test with Bonferroni correction. Dichotomous variables were compared using the Chi-square test. Molecular predictors of TOD were assessed in a stepwise regression analysis. The dependent variables were absolute, and standardized values of cIMT and variables which correlated with TOD or differentiated patients with or without TOD were chosen as independent variables. Statistical analysis was done using SPSS12.0PL software (IBM Corp., Armonk, NY).

Calculations to determine whether observed genotype frequencies were consistent with the Hardy–Weinberg equilibrium, which means that in a population in the absence of disturbing factors they will remain constant from one generation to the next, were performed with the Court online calculator (http://www.tufts.edu/~mcourt01/Documents/Court%20lab%20-%20HW%20calculator.xls).* P* values of <0.05 were regarded as statistically significant, and those ranging between 0.05 and 0.1 were regarded as a statistical tendency.

## Results

### Clinical and biochemical phenotype

Children with PH had significantly greater BMI, higher serum UA, hsCRP, homocysteine, lipoprotein a, TTG/HDL ratio, plasma insulin, HOMA-IR, ADMA and oxyLDL and lower GPx activity and GSH concentration than normotensive children. They also had significantly greater cIMT values than healthy children. Children with PH had higher triglyceride concentrations and a lower birth weight than normotensive children, but the difference was non-significant for both parameters (Tables 1, 2).

**Table 1:** Demographic, clinical and genetic data in patients with primary hypertension and control group

Variable	Control group,* N* = 83 (*n* = 40 boys)	PH patient group,* N* = 126 (*n *= 95 boys)	*p*
Age (years)	12 (5–18)	15 (5–18)	ns
Birth weight (g)	3520 (1960–4400)	3355 (1410–5250)	0.06
BMI (kg/m^2^)	19.2 ± 3.9	24.8 ± 4.5	<0.001
BMI SDS	0.27 ± 1.13	1.75 ± 1.7	<0.001
WC (cm)	n.a.	84 (53–111)	-
WC SDS	n.a.	1.62 (−2.03 to 6.12)	-
cIMT (mm)	0.40 (0.31–0.53)	0.43 (0.34–0.63)	<0.001
cIMT SDS	0.50 (−2.27 to 3.46)	0.97 (−1.37 to 7.83)	<0.001
LVMi (g/m^2.7^)	n.a	35.3 (19.1–62.3)	-
Number of patients with GT and TT alleles	45 (54.2 %)	66 (52.4 %)	ns
Number of patients with TT alleles	8 (9.6 %)	13 ( 10.3 %)	ns

Data are presented as the mean ± standard deviation (SD), as the median with the range in parenthesis or as a number with the percentage in parenthesis, as appropriate

*PH* Primary hypertension, *BMI* body mass index, *cIMT* carotid intima-media thickness, *LVMi* left ventricular mass index, *SDS* standard deviation score, *n.a*. not assessed, *GT* carriers of G894 and T894 allele, *TT* T894 homozygotes, *WC* waist circumference

**Table 2:** Laboratory data for patients with primary hypertension and the control group

Variable	Contro group (*N* = 83)	PH patient group (*N* = 126)	*p*
Cholesterol (mg/dl)	175.6 ± 39.1	173.6 ± 33.7	ns
Triglicerydes (mg/dl)	69 (38–312)	84 (28–425)	0.05
HDL-cholesterol (mg/dl)	46.6 ± 9.8	44.6 ± 9.6	ns
TTG/HDL	1.48 (0.64-15)	1.84 (0.58–42)	0.01
LDL-cholesterol (mg/dl)	111 (60–185)	110 (43–226)	ns
Lp(a) (mg/dl)	11.7 ± 7.6	15.5 ± 11.7	0.02
Uric acid (mg/l)	4.08 ± 0.63	5.48 ± 1.19	<0.001
Homocysteine (μmol/l)	8.9 ± 3.4	10.2 ± 3.5	0.02
hsCRP (mg/dl)	0.44 (0.12–6.74)	0.74 (0.02–4.68)	0.01
Fasting glucose (mg/dl)	90 (78–105)	85 (67–109)	0.001
Fasting insulin (mU/ml)	9 (3.3–15)	12.8 (3–39)	0.001
HOMA-IR	1.9 (0.74–3.28)	2.78 (1.11–8.77)	0.001
GPx (U/gHb)	32.8 (20.1–42.1)	31.5 (23.8–39.9)	0.005
GSH (μmol/l)	792.5 (253.2–889.1)	760.1 (441.7–889.4)	0.001
ADMA (μmol/l)	0.46 (0.15–1.30)	0.55 (0.25–1.45)	0.02
oxyLDL (mU/ml)	260.9 (0.21–1272)	400.15 (45.8–1372)	0.008
Albuminuria(mg/24 h)	n.a.	14.7 (2–270)	

Data are presented as the mean ± SD or as the median with the range in parenthesis, as appropriate

*HDL* high-density lipoprotein-cholesterol, *TTG* total triglycerydes, *LDL* low-density lipoprotein-cholesterol, *Lp*(*a*) lipoprotiein a, *hsCRP* high-sensivity C-reactive protein, *HOMA-IR* homeostasis model assessment for insulin resistance, *GPx* glutathione peroxidase, *GSH* reduced glutathione, *ADMA* serum asymmetric dimethyloarginine, *oxyLDL* oxidized LDL-cholesterol, *ns* not significant

In the PH group, 92 (73 %) children had ambulatory hypertension and 34 (27 %) children had severe ambulatory hypertension. MS was diagnosed in 22 (17.5 %) patients and in none of the controls. Also in this patient group, 39 (31 %) children had LVH, 21 (16.6 %) children had cIMT of >2 SDS and 18 (14.3 %) children had albuminuria of >30 mg/24 h.

We found a significant correlation between the LVMi and all of the assessed anthropometrical parameters [BMI (*r* = 0.34), BMI-SDS (*r* = 0.38), WC (*r* = 0.39), WC-SDS (*r* = 0.34) and WHR (*r* = 0.37)]. The cIMT-SDS correlated with SBP/24 h (*r* = 0.19, *p* < 0.05), hsCRP (*r* = 0.25, *p* < 0.05), GPx (*r* = 0.23, *p* < 0.05) and albuminuria (*R* = 0.21, *p* < 0.05). Positive correlations were found between the LVMi and values of albuminuria and hsCRP (*R* = 0.22, *p* < 0.05) and a negative correlation was found with birth weight (*R* = −0.27, *p* < 0.05).

Compared to hypertensive children with a cIMT of <2 SDS, hypertensive children with cIMT of >2 SDS presented significantly higher values of SBP/24 h (134 ± 9 vs. 128 ± 8 mmHg; *p* < 0.05), SBPi/24 h (1.05 ± 0.07 vs. 1.01 ± 0.06; *p* < 0.05) and albuminuria [24.2 (4.7–270) vs. 13.3 [2–172] mg/24 h; *p* < 0.05].

In the control group, cIMT-SDS trended to correlate with hsCRP (*R* = 0.21, *p* = 0.06) and fasting glucose (*R* = 0.24, *p* = 0.07). No significant differences between controls with a cIMT of >2 SDS (12 patients, 14.5 %) and those with a cIMT of <2 SDS (71 patients , 85.5 %) were found.

### Molecular studies—baseline comparisons

The frequency of the T allele was 52.4 % in the PH group and 54.2 % in the control group [not significant (n.s.)], and in both groups observed genotype frequencies were consistent with the Hardy–Weinberg equilibrium ( PH: chi^2^ = 0.06, *p* = 0.798, controls: chi^2^ = 0.054, *p* = 0.8159).

Hypertensive T allele carriers had greater cIMT, cIMT-SDS (both significant at *p* < 0.05) and albuminuria (n.s. at *p* = 0.1) than G allele carriers but did not differ in relation to hypertension severity and LVMi (Table 3). No significant correlations between the assessed parameters of oxidative stress and anti-oxidative defense and *eNOS* G894T gene polymorphism were found. T and G allele distribution did not differ between patients with and without MS [TT frequency: 3 (13.6 %) patients vs. 10 (9.6 %) patients, respectively; GG: 10 (45.5 %) patients vs. 50 (48.1 %) patients, respectively; not significant].

**Table 3:** Demographic, clinical and laboratory data in patients with primary hypertension according to *eNOS* G894T genotype variants

Variables	GG (1)	GT + TT (2)	GT (2a)	TT (2b)	*p*
*N*	60	66	53	13	
Birth weight (g)	3325 (1790–4200 )	3400 (1410–5250)	3300 ( 1410–4000)	3500 (3140–5250)	1 vs. 2b *p* = 0.03 2a vs. 2b *p* = 0.06
BMI	24.9 ± 4.4	24.8 ± 4.6	24.6 ± 4.2	25.6 ± 6.3	ns
BMI-SDS	1.55 (−1.26 to 6.03)	1.64 (−0.81 to 9.69)	1.64 (−1.56 to 9.69)	1.79 (−0.81 to 5.8)	ns
WC (cm)	85.14 ± 10.8	81.23 ± 12.9	81.23 ± 12.08	81.33 ± 16.6	ns
WC-SDS	1.82 (−0.94 to 4.88)	1.38(−2.03–6.1)	1.35(−2.03–6.12)	1.40 (−1.1 to 4.5)	ns
WHR	0.86 ± 0.07	0.83 ± 0.06	0.82 ± 0.07	0.84 ± 0.07	1 vs. 2 *p* = 0.01 1 vs. 2a *p* < 0.01
SPBP/24 h (mmHg)	129 ± 8	130 ± 8	130 ± 8	130 ± 9	ns
SBPi/24 h	1.02 (0.89–1.17)	1.02(0.91–1.23)	1.02 (0.92–1.20)	1.03 (0.90–1.23)	ns
DBP/24 h (mmHg)	73 ± 6	72 ± 8	72 ± 7	73 ± 9	ns
DBPi/24 h	0.95 (0.81–1.23)	0.94 (0.69–1.27)	0.94 (0.82–1.27)	0.94 (0.8–1.12)	ns
HR/24 h (/min)	82 ± 10	77 ± 12	77 ± 12	78 ± 10	1 vs. 2 *p* = 0.02 1 vs. 2a *p* = 0.03 1 vs. 2b *p* = 0.1
IMT (mm)	0,43 (0.34–0.52)	0.44 (0.345–0.62)	0.43 (0.345–0.62)	0.465 (0.36–0.57)	1 vs. 2 *p* = 0.01 1 vs. 2a *p* = 0.05 1 vs. 2b *p* = 0.02
IMT-SDS	0,86 (−1.05 to 3.12)	1.03 (−1.37 to 7.2)	1 (−1.37–7.2)	1.49 (−0.35 to 2.7)	1 vs. 2 *p* = 0.03 1 v.s 2a *p* = 0.06 1 vs 2b *p* = 0.03
Albuminuria (mg/24 h)	13.5 (2–57.6)	15.2 (4.6–250)	14.7 (4.6–250)	16.2 (9.3–54.6)	1 vs. 2 *p* = 0.17
LVMi (g/m^2.7^)	35.2 ± 8.0	36.9 ± 9.4	36.6 ± 9.4	37.7 ± 9.6	ns
RWT (mm)	0.32 (0.23–0.59)	0.36 (0.23–0.59)	0.35 (0.23–0.59)	0.36 (0.28–0.45)	1 vs. 2b *p* = 0.1
TTG/HDL	1.87 (0.78–-12.9)	1.84 (0.58–42.5)	1.9 (0.58–42.5)	1.77 (0.78–2.91)	ns
hsCRP (mg/l)	0.76 (0.02–3.98)	0.69 (0.05–4.68)	0.84 (0.05–4.68)	0.41 (0.14–0.82)	1 vs. 2b *p* = 0.05 2a vs. 2b *p* = 0.06
Fasting insulin (mU/ml)	12.9 (6.4–39)	12.8 (3–36)	12.8 (3–36)	12.9 (6.7–30)	ns
HOMA -R	2.86 (1.26–8.77)	2.68 (1.1–7.9)	2.73 (1.1–7.92)	2.64 (1.38–6.23)	ns
HbA1c (%)	5.4 (4.1–6.8)	5.3 (4.2–6.6)	5.3 (4.2–6.6)	5.0 (4.5-5–6)	1 vs. 2 *p* = 0.11 1 vs. 2b *p* = 0.1
GPx (U/gHb)	31.6 (28.3–39.9)	31.4 (23–8-38.1)	31.3 (23.8–38.1)	33.2 (30.8–36.9)	2a vs. 2b *p* = 0.07
Number of patients with MS	10	12	9	3	ns

Data are presented as the mean ± SD, as the median with the range in parenthesis or as a number, as appropriate

*GG* G894 homozygotes, *GT* carriers of G894 and T894 allele, *TT* T894 homozygotes, *WHR* waist-to-hip ratio, *SBP/24 h* mean systolic blood pressure in 24-h ambulatory blood pressure monitoring (ABPM), SBPi/24 h index of SBP/24 h, *DBP/24 h* mean diastolic systolic blood pressure in 24-h ABPM, *DBPi/24 h* index of DBP/24 h, *HR/24 h* mean heart rate in 24-h ABPM, *RWT* relative wall thickness, *HbA1c* glycated hemoglobin, *MS* metabolic syndrome

In comparison to GG homozygotes, TT homozygotes with PH had higher cIMT [0.465 (0.36–0.57) vs. 0.43 (0.34-0.52) mm; *p* = 0.02], cIMT-SDS [1.49 (−0.35 to 2.7) vs. 0.86 (−1.05 to 3.12); *p* = 0.03] and a tendency to higher relative wall thickness (RWT) [0.36 (0.28–0.45) vs. 0.32 (0.23–0.59) mm; *p* = 0.1]. GG homozygotes had a significantly lower birth weight, higher visceral fat accumulations, higher hsCRP and higher heart rate in ABPM than the other groups (Table 3).

In the control group no significant correlations between assessed parameters and the *eNOS* G894T gene polymorphism were found. However, T allele carriers tended to have greater cIMT than GG homozygotes [0.41(0.33-0.53) mm vs. 0.40 (0.31 -0.48) mm; *p* = 0.09).

TT homozygotes with PH in comparison to TT homozygotes in the control group presented only significantly higher values of cIMT (*p* = 0.04) and a tendency to higher cIMT SDS (*p* = 0.07) (Table 4).

**Table 4:** Comparison of some clinical and laboratory data of TT allele carriers in the control and primary hypertension group

Variables	TT controls	TT patients with PH	*p*
Number of pts (%)	8 (9.6 %)	13 (10.3 %)	ns
BMI (kg/m^2^)	20.7 ± 3.7	25.6 ± 6.3	0.07
BMI-SD	0.62 ± 0.98	1.89 ± 2.05	ns
cIMT (mm)	0.41 (0.31–0.44)	0.46 (0.37–0.57)	0.04
cIMT-SDS	0.67 (−1.58 to 1.72)	1.49 (−0.35 to 2.52)	0.07
hsCRP (mg/l)	0.24 (0.15–0.71)	0.41 (0.14–0.82)	ns
TTG/HDL	1.8 (1.0–2.9)	1.8 (0.8–2.9)	ns
Fasting insulin (mU/ml)	12 (7.6–12)	12.9 (6.7–30)	0.1
HOMA-IR	2.42 (1.06–2.85)	2.64 (1.38–6.23)	ns

Data are presented as the mean ± SD, as the median with the range in parenthesis or as a number with the percentage in parenthesis, as appropriate

*BMI* body mass index, *cIMT* carotid intima-media thickness, *hsCRP* high-sensivity C-reactive protein, *TTG/HDL* total triglycerydes/ high density lipoprotein cholesterol, *HOMA-IR* homeostasis model assessment for insulin resistance

Stepwise regression analysis revealed that the main predictors of increased cIMT-SDS (*R*^2^ = 0.23,* F* = 1.8) were the G894T gene polymorphism (beta = 0.342, *p* = 0.01) and GPx activity (beta = 0.288, *p* = 0.047). For absolute cIMT values (*R*^2^ = 0.22,* F* = 1.97) the main predictors were also G894T gene polymorphism (beta = 0.343, *p* = 0.01) and GPx activity (beta = 0.28, *p* = 0.049).

## Discussion

The main finding of our study is that the T894 allele of the *eNOS* gene is associated with early vascular damage in children with PH independently of metabolic abnormalities. This association was not found in normotensive children.

Reports showing an association between the G894T polymorphism, vascular complications and hypertension in humans are not consistent [10–17, 36]. The meta-analysis of Niu and Qi indicated that the T894 allele may be associated with an increased risk of hypertension. However, this association exhibited no significance in white people, suggesting the heterogeneous associations of G894T polymorphism in specific populations (Asian populations) [21]. Similarly, inter-ethnic differences in the distribution of *eNOS* genetic variants have been described in various studies, including comparisons of black and white Brazilians and of Caucasians, Afro-Americans and Asians [20, 37]. Thus, our findings may be limited only to a population of Caucasian children.

In our homogenous group of Caucasian children the frequency of the T allele was 52.4 % in the PH group and 54.2 % in the control group; this slight difference between the groups was not significant. Also, we did not find any differences in BP levels or prevalence of MS between the analyzed genotypes. These results are similar to those of Miranda at al. who did not find any association between the G894T polymorphism and MS in obese children and adolescents [28]. However, these authors indicated that the CC genotype for the T786C polymorphism of *eNOS* is associated with MS [28].

In our study, hypertensive T allele carriers had greater cIMT and tended to have greater albuminuria that the G allele carriers (Table 3). Moreover, in comparison to the GG homozygotes, the TT homozygotes presented with higher birth weight, lower visceral fat accumulation, lower hsCRP and lower heart rate in ABPM, a significantly higher cIMT and a tendency to greater RWT. Similarly, in the control group, T allele carriers tended to have greater cIMT. The correlation between T allele frequency and intima-media thickening has also been found in some groups of adult patients [14–16]. Some authors have reported that the 894 T allele of the *eNOS* polymorphism is also associated with carotid atheroma and with the presence, extent and severity of angiographically assessed coronary artery disease [12, 13]. Czarnecka et al. found higher cIMT values in both hypertensive T allele carriers and among T allele carrier offspring of hypertensive patients [15]. Our finding of an association between the 894 T allele and greater cIMT only in hypertensive children suggests that genetic polymorphism of the *eNOS* gene predisposes to arterial injury only when the arterial wall is exposed to higher blood pressure. However, it does not mean that the *eNOS* gene polymorphism is associated with elevated blood pressure.

Arterial remodeling in HT patients is mainly determined by reduced NO bioavailability. Apart from the significance of the genetically conditioned decreased eNOS release/availability, obesity and inflammation also lead to reduced eNOS bioavailability and consequently to cardiovascular diseases [26, 38, 39].

Jiménez-Morales found that their subjects with the TT genotype displayed a lower vascular response (lower increase in post-ischemic capillary flow) compared with TG and GG genotypes and that this response was improved after an intake of meals rich in high-phenol virgin olive oil [40]. Similarly, Leeson et al. found a positive relationship between n-3 fatty acid level and flow-mediated dilation in 894 T carriers, but not in G894 homozygotes. Additionally, among men, smoking has been associated with lower flow-mediated dilation in T allele carriers but not in GG homozygotes [17]. A study of the functional consequences of the Glu298Asp polymorphism of the *eNOS* gene in healthy volunteers found that TT allele carriers, i.e. Asp homozygotes, had decreased vasodilatory response to acetylcholine in the forearm, which indicates blunted endothelial-dependent vasodilation [41]. The role of the Glu298Asp polymorphism in the regulation of blood pressure status was recently evaluated in over 2000 children and adolescents participating in the European Youth Heart Study. Asp homozygotes were found to have slightly higher blood pressure values at rest compared to Glu298 carriers. Interestingly, this difference was found only in adolescents (pubertal and post-pubertal subjects), but not in prepubertal children. Moreover, physical activity modified the genetic effect, which was most apparent in inactive subjects [27].

It is suspected that the Glu298Asp polymorphism reduces the degree of interaction of eNOS with caveolin-1 and thereby hinders the localization of eNOS in caveolae and diminishes shear-dependent eNOS activation [16, 42]. However, the molecular mechanism of the effects of different *eNOS* polymorphisms is not clear. In studies not limited to the analysis of SNPs but which analyzed the effects of different haplotypes of *eNOS*, such as a SNP in the promoter region (T^−789^C), a variable tandem repeat in intron 4 and a Glu298Asp SNP in intron 7, subjects who had haplotype “C-4b-Glu” had the lowest plasma and whole blood nitrite levels [43, 44]. Interestingly, there marked inter-ethnic differences in the distribution of different haplotypes have been observed [37]. Souza-Costa at al. compared the distribution of some *eNOS* haplotypes in normotensive obese/hypertensive obese children with healthy controls and found that only the CbG haplotype constellation was more frequent in hypertensive obese children in comparison to the other analyzed group [26].

Following these observations, the eNOS modulatory role of statins and virgin olive oil may have an influence on functional regulation of the cardiovascular system and may offer new perspectives for the better use of statins and phenol-rich olive oil in ameliorating cardiovascular disorders, especially in patients with downregulated eNOS function, such as carriers of the TT allele of the G894T polymorphism. Moreover, the interaction between the genetic variation in *eNOS* and BP may also be modified by physical activity. Physical activity may strengthen the production and effect of NO in the regulation of BP through endothelial vasodilatation and could be an effective way of controlling BP in individuals with a genetic predisposition toward hypertension or hypertensive children with subclinical arterial injury [27, 45].

The main limitation of our study was the low number of patients and controls. Because of this, some differences between groups were not statistically significant. It is important to emphasize that some authors have failed to find any relationship between the 894 T variant and the risk of atherosclerosis [18, 46]. In the systematic assessment and meta-analyses of candidate gene polymorphisms studied in more than 5000 subjects, including the most extensively studied polymorphisms for cIMT, the apolipoprotein E ε2/ε3/ε4 polymorphism is the only one to date for which a convincing association with cIMT has been demonstrated [47]. Even if the number of patients included in our study is small, it should be emphasized that all participants were clinically well-defined and selected. Our study is the first to show an association between the *eNOS* G894T polymorphism and early vascular damage in children with untreated PH. Certainly, we have examined only one selected polymorphism, which provides less verified information. PH is a complex state that involves many genes apart from the *eNOS* gene and is not entirely explained by genetic factors. Other risk factors may modify the effects of *eNOS* polymorphisms on the risk of vascular damage.

In conclusion, we demonstrate for the first time in children with PH that the G894T polymorphism of the *eNOS* gene is associated with early vascular damage independent of metabolic abnormalities.
